# Lower Gene Expression of Angiotensin Converting Enzyme 2 Receptor in Lung Tissues of Smokers with COVID-19 Pneumonia

**DOI:** 10.3390/biom11060796

**Published:** 2021-05-26

**Authors:** Francesca Lunardi, Francesco Fortarezza, Luca Vedovelli, Federica Pezzuto, Annalisa Boscolo, Marco Rossato, Roberto Vettor, Anna Maria Cattelan, Claudia Del Vecchio, Andrea Crisanti, Paolo Navalesi, Dario Gregori, Fiorella Calabrese

**Affiliations:** 1 Department of Cardiac, Thoracic, Vascular Sciences and Public Health, University of Padova Medical School, 35128 Padova, Italy; francesca.lunardi@unipd.it (F.L.); francescofortarezza.md@gmail.com (F.F.); luca.vedovelli@unipd.it (L.V.); federica.pezzuto@phd.unipd.it (F.P.); dario.gregori@unipd.it (D.G.); 2 Department of Medicine, University of Padova Medical School, 35128 Padova, Italy; annalisa.boscolobozza@aopd.veneto.it (A.B.); marco.rossato@unipd.it (M.R.); roberto.vettor@unipd.it (R.V.); annamaria.cattelan@aopd.veneto.it (A.M.C.); paolo.navalesi@unipd.it (P.N.); 3 Department of Molecular Medicine, University of Padova Medical School, 35121 Padova, Italy; claudia.delvecchio@unipd.it (C.D.V.); andrea.crisanti@unipd.it (A.C.)

**Keywords:** SARS-CoV-2, COVID-19, angiotensin converting enzyme receptor, smoking

## Abstract

Angiotensin-converting enzyme 2 (ACE-2) is the main cell entry receptor for severe acute respiratory syndrome-Coronavirus-2 (SARS-CoV-2), thus playing a critical role in causing Coronavirus disease 2019 (COVID-19). The role of smoking habit in the susceptibility to infection is still controversial. In this study we correlated lung ACE-2 gene expression with several clinical/pathological data to explore susceptibility to infection. This is a retrospective observational study on 29 consecutive COVID-19 autopsies. SARS-CoV-2 genome and ACE-2 mRNA expression were evaluated by real-time polymerase chain reaction in lung tissue samples and correlated with several data with focus on smoking habit. Smoking was less frequent in high than low ACE-2 expressors (*p* = 0.014). A Bayesian regression also including age, gender, hypertension, and virus quantity confirmed that smoking was the most probable risk factor associated with low ACE-2 expression in the model. A direct relation was found between viral quantity and ACE-2 expression (*p* = 0.028). Finally, high ACE-2 expressors more frequently showed a prevalent pattern of vascular injury than low expressors (*p* = 0.049). In conclusion, ACE-2 levels were decreased in the lung tissue of smokers with severe COVID-19 pneumonia. These results point out complex biological interactions between SARS-CoV-2 and ACE-2 particularly concerning the aspect of smoking habit and need larger prospective case series and translational studies.

## 1. Introduction

Coronavirus disease 2019 (COVID-19) has affected approximately 120 million people in a devastating worldwide pandemic [1]. The disease is characterized by an extremely heterogeneous clinical course, ranging from asymptomatic cases or cases with mild flu-like symptoms to the most severe cases of bilateral interstitial pneumonia requiring hospital admission or even intensive care unit (ICU) treatment [2].

In the early stage of the outbreak, genomic analysis studies and pairwise protein sequence analysis revealed that a SARS-related coronavirus, the SARS-CoV-2 (severe acute respiratory syndrome-Coronavirus-2), was the causative pathogen agent. The full-length genome sequencing of the virus showed that the sequences share a 79.6% identity to SARS-CoV, responsible for the 2003 SARS epidemic. Rapid global spread and transmission of COVID-19 provides the virus with substantial opportunities for the natural selection of rare but favorable mutations. Indeed, many mutations on the spike (S) protein seem to be favorable for viral entry, survival capability and transmission [3,4]. It was found that SARS-CoV-2 uses the same functional host receptor as SARS-CoV, the angiotensin-converting enzyme 2 (ACE-2), through binding to the viral S protein [5]. Following SARS-CoV-2 binding with ACE-2, the entry of the virus into the cell requires the priming and cleavage of protein S by a serine protease, TMPRSS2, allowing the fusion of the viral and cell membranes [6].

ACE-2 is a membrane-associated carboxypeptidase that catalyzes the conversion of angiotensin I (a vasoconstrictor) to the nonapeptide angiotensin (1–9) and of angiotensin II in the heptapeptide angiotensin (1–7) (vasodilators), playing a fundamental role in the regulation of blood pressure and in the renin–angiotensin–aldosterone system (RAAS). Its expression in human tissue is almost ubiquitous, including cardiovascular tissue, the respiratory tract, small intestine, kidneys, and central nervous system [7].

One of the hallmarks of COVID-19 is the presence of a variable degree of vascular injury mostly in the form of micro/macro-thrombosis, endothelialitis and neoangiogenesis [8,9], likely related to the virus’ spread into endothelial cells, which show ACE-2 receptors as well as other cell types.

These findings indicate that organs with a high ACE-2-expressing cell number should be considered to be at high risk for SARS-CoV-2 infection [10].

In the lung, ACE-2 is expressed in the airway epithelia as well as in several cell types of lung parenchyma. The immunofluorescence studies have shown that ACE-2 protein was abundantly expressed on the apical surface of well-differentiated and polarized airway epithelia [11], in type II pneumocytes [12], alveolar macrophages [13], and lung endothelial cells [14]. However, the exact role of ACE-2 as a mediator of lung disease severity remains to be determined. Mice engineered to express high levels of human ACE-2 succumbed to infections with the SARS coronavirus more quickly than mice expressing low levels of human ACE-2, suggesting that increasing ACE-2 enhances viral entry susceptibility [10]. At the same time, it has been shown that ACE-2-knockout mice are vulnerable to a variety of lung injuries, and ACE-2 expression has been reported to play a protective role in the respiratory tract [15]. As ACE-2 expression is both necessary and sufficient for SARS-CoV-2 infection [16], it seems highly likely that an expansion of ACE-2 positive cells in the lungs would facilitate viral dissemination, but it remains possible that the presence of ACE-2 has some beneficial functions as well.

Many of these studies have been carried out in animal models and in vitro always from healthy or non-infected lung tissues [17]. The expression of ACE-2 in tissues coming from COVID-19 patients has been previously reported although not in the lung [18]. Moreover, studies of correlations between tissue viral receptors and major clinical-pathological data are still largely missing. There are several studies that have hypothesized altered ACE-2 expression by correlating the severity and progression of COVID-19 with many different variables such as age, sex, ethnicity, drugs, various comorbidities and smoking [19] sometimes reporting conflicting results, as in the case of smoking status [20]. Indeed, several case series have highlighted that active smoking status was significantly lower in COVID-19 patients, while others found that smoking was an independent risk factor for a more severe disease and a worse prognosis [21,22,23,24,25], implying a connection between an altered expression of ACE-2 and cigarette smoke. 

In this interesting and conflicting scientific debate, molecular studies evaluating the mechanisms leading to altered ACE-2 expression in COVID-19 patients are still lacking.

Accordingly, we aimed to analyze lung ACE-2 gene expression and correlate it with several clinical/pathological data of patients with severe COVID-19 pneumonia to determine possible susceptibility to infection.

## 2. Materials and Methods

### 2.1. Study Population

This retrospective observational study included 29 consecutive COVID-19 laboratory-confirmed autopsies performed at the University Hospital of Padua from 23 March to 10 October 2020, according to national and international protocols [26]. The Ethics Committee of our Center was informed about our study, which complied with the Declaration of Helsinki. The diagnosis of COVID-19 was made according to the WHO interim guidance [27]. Specifically, nasopharyngeal swabs were tested by sequencing or real-time polymerase chain reaction (PCR) according to international standards. For each patient, the following demographic data and clinical characteristics were recorded: age, gender, comorbidities (in particular cardiovascular), other respiratory pathogens, duration of the disease, and smoking history.

Autoptic examinations were carried out with a postmortem interval ranging from 24 hours to 6 days. Lungs were evaluated as previously described [28]. Briefly, 16 tissue blocks from airways and each lung (3 blocks/lobe) were sampled. Based on the presence and severity of some histological parameters, each patient was categorized by expert pathologists (FC, FF, FP) in a prevalent histological phenotype, as previously described [29].

Pathological alveolar injury (AI) prevalent phenotype was defined when the combined score of hyaline membranes, organizing pneumonia, pneumocyte type 2 hyperplasia, and squamous metaplasia were found to be at least two times greater than vascular alterations. These lesions, including microthrombi, large thrombi, vasculitis, and capillary inflammation, identified the prevalent vascular injury (VI) phenotype, and a prevalent vascular phenotype when VI scores were double AI scores. A mixed phenotype was defined when lesions of both AI and VI were equally present, and a prevalent phenotype was unremarkable.

### 2.2. Molecular Analyses

Total RNA was extracted from about 30 mg of frozen lung autopsy tissues using a RNeasy Plus Mini Kit (Qiagen, Hilden, Germany) with a sample homogenization in a Tissue Lyzer System (Qiagen, Hilden, Germany) according to the manufacturer’s instructions. Quantity and quality of prepared RNA were examined by Nanodrop One/OneC Microvolume UV-Vis Spectrophotometer (ThermoFischer Scientific, Waltham, MA, USA). The cDNA was synthesized from 500 ng of total RNA, using 50 μM Random Hexamers (Invitrogen, ThermoFischer Scientific, Watham, MA, USA), 10 mM dNTPs mix (Invitrogen, ThermoFischer Scientific, Waltham, MA, USA), RNase Inhibitor 20 U/µL (Applied Biosystems, ThermoFischer Scientific, Waltham, MA, USA), 50 U/µL of Multi Scribe Reverse Transcriptase (Applied Biosystems, Thermo Fisher Scientific, Waltham, MA, USA) in a final volume of 20 uL following the manufacturer’s protocol. The cDNA quality was confirmed by the amplification of housekeeping gene glyceraldehyde3-phosphate dehydrogenase (GAPDH). Quantitative determination of mRNA levels of the ACE-2 gene was performed in triplicate on a Light Cycler 480 II (Roche Applied Science, Mannheim, Germany) using SYBR Green based quantification. Reactions were prepared in a final volume of 20 µL with 1× SYBR Green PCR Master Mix (Applied Biosystems, Thermo Fisher Scientific, Waltham, MA, USA), 5 µM of forward and reverse primers and 5 µL of cDNA in 45 cycles of amplification. The used oligonucleotide primers were: ACE-2 forward 5′-CATTGGAGCAAGTGTTGGATCTT-3′; ACE-2 reverse 5′-GAGCTAATGCATGCCATTCTCA-3′; GAPDH forward 5′-AAGGTGAAGGTCGGAGTCAA-3′; and GAPDH reverse 5′-ACCAGAGTTAAAAGCAGCCC-3′. The reaction conditions were 10 min of denaturation and enzyme activation at 95 °C followed by denaturation at 95 °C for 10 seconds, annealing at 58 °C for 40 s, and extension and florescence acquiring at 60 °C for 30 s. GAPDH was used as an internal control to normalize the target genes. For each sample, three replicates were performed, and the average Ct was determined and then the ΔCt value was calculated by normalizing target genes (ACE-2) with the housekeeping gene GAPDH. Normal lung tissue samples coming from five lung donors were used as controls. The relative transcript levels (fold-changes) were calculated as x = 2^−ΔΔCt^ in which ΔΔCT = ΔCt (SARS-CoV-2 positive patients) − ΔCt (lung donors).

Quantitative determination of SARS-CoV-2 was also performed by using the CDC 2019-Novel Coronavirus (2019-nCoV) real-time RT-PCR Diagnostic Panel [30].

**Figure 1:**
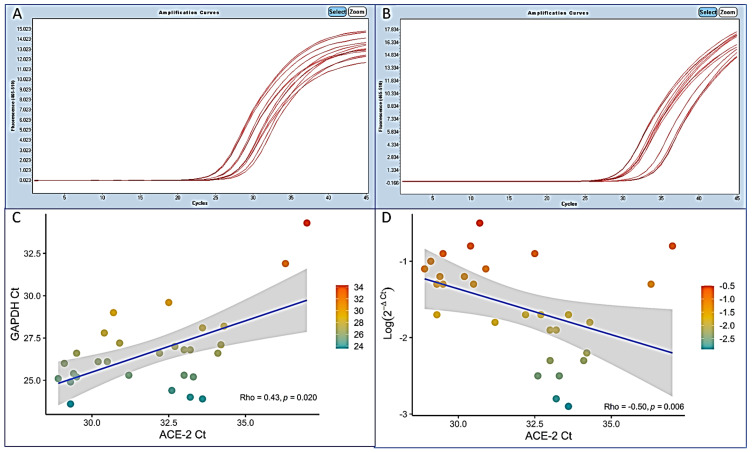
Real-Time PCR for ACE-2 expression. Typical amplification plots of GAPDH (**A**) and ACE-2 (**B**) genes. Cts of GAPDH plotted against the ACE-2 gene Cts (Rho = 0.43, *p* = 0.020) (**C**). Normalized ACE-2 Ct values (log(2^−ΔCt^) = log(2 − (Ct_target_ − Ct_reference_)) plotted against the ACE-2 gene Cts (**D**). The normalized Ct values are relative expression values (ratios of target gene to housekeeping gene) and are transformed to logarithmic scale for graphical representation. Shaded areas represent the 95% CI of the linear correlation line.

The reaction conditions were 10 minutes at 50 °C, then 95 °C for 3 min, followed by 45 cycles of denaturation at 95 °C for 3 sec, then annealing and extension at 55 °C for 30 s. To correct for sampling variability, we used the human RNAse P as a reference to normalize the viral load by the comparative Ct method (ΔCt), that transforms the Ct into relative loads (ratios of viral target to human target). Figure 1 shows a plot of SARSCoV-2 gene N1 Ct normalized with the human RNAse P Ct values against the gene N1 Ct. The plot shows an inverse linear correlation, which is expected because Ct values indeed reflect viral loads, but the dispersion of the data may reach up to four log units (ten thousand-fold) for any given Ct (black arrow). 

### 2.3. Statistical Analysis

All clinical and pathological data were recorded in an electronic database. Data are presented as medians (with first and third quartile) for continuous variables and as percentages for categorical variables. Considering age, patients were divided on the basis of the median value (82 years old) into “older” (≥82 years old) and “younger” (<82 years old). All statistics were performed with ΔCt values, that are inversely related to the expression of the target gene. Based on median ACE-2 ΔCt value, patients were divided in “high expressors” (ΔCt < 5.6) or “low expressors” (ΔCt ≥ 5.6) and compared for all available clinical/morphological data. Wilcoxon rank sum test, Pearson’s Chi-squared test, Fisher’s exact test, and Wilcoxon rank sum exact test were used to compare variables in the two groups (smokers vs. non-smokers and high vs. low expressors). False discovery rate correction for multiple testing (*q*-value) was implemented. Linear correlations were explored with the Spearman’s Rho coefficient. 

We fitted a Bayesian general linear model (student family with an identity link) (estimated using Markov Chain Monte Carlo sampling with 4 chains of 2000 iterations and a warmup of 500) to predict ACE-2 expression with smoker (yes/no), age, gender, hypertension (yes/no), and virus quantity. Priors over parameters were all set as student t (location = 3, 0, scale = 1.00) distributions. Following the Sequential Effect eXistence and sIgnificance Testing (SEXIT) framework, we reported the median of the posterior distribution and its 95% CI (Highest Density Interval), along the probability of direction (pd), the probability of significance and the probability of being large. The thresholds beyond which the effect is considered as significant (i.e., non-negligible) and large are |0.05| and |0.30|. Convergence and stability of the Bayesian sampling has been assessed using R-hat, which should be below 1.01, and effective sample size (ESS), which should be greater than 1000. Analysis and graph were produced with the R software v.4.0 [31] using the tidyverse, stats, brms, gtsummary, ggstatsplot and report packages.

## 3. Results

### 3.1. Study Population

Table 1 reports the main clinical characteristics of the study population.

Patients had a median (IQR) age of 82 (75–87) years and consisted mainly of males (59%). Information about smoking status was carefully collected and turned out to be available in 26/29 patients. In particular, 10 of them were smokers (38.5%) (7 former and 3 current smokers) and 16 were non-smokers (61.5%). Median disease duration (calculated from the onset of symptoms to death) was 9 days, ranging from 3 to 54. Comorbidities were present in 28/29 patients (97%) and 25 of them were affected by cardiovascular diseases. Most patients presented a minimum of two to a maximum of six comorbidities. The most frequent comorbidities were hypertension (17), type II diabetes (7), dementia (7), atrial fibrillation (5), ischemic heart disease/heart failure (5), chronic obstructive pulmonary disease (5), neoplasia (5), and vasculopathy (4). Dyspnea was the most frequent symptom and was present in 90% of patients.

After a systematic morphological evaluation, histology showed heterogeneous features: 45% of patients showed a prevalent pattern of vascular injury, while 14% of them had an acute lung injury and 41% a mixed phenotype.

### 3.2. Molecular Analyses

RNA was extracted from frozen lung tissue samples obtained from all patients and from normal lung tissue samples coming from five non-implanted donor lungs (three females, two males, mean ± SD age: 49.2 ± 7.7 years, all non-smokers), used as “healthy” controls. Quantity and quality of RNA samples were adequate for the real-time polymerase chain reaction (PCR) analysis. In particular, mean (±SD) RNA concentration was 236 ± 185; mean (±SD) A260/280 and A260/230 ratios were 2.09 ± 0.03 and 1.54 ± 0.53, respectively.

In all patients, the positivity for SARS-CoV-2 was confirmed in lung tissues by real-time PCR, showing median (Q1–Q3) values of N1 ΔCt of 2.9 (−3.4–6.8), ranging from −9.3 to 13.7. Donor lungs were completely negative.

Real-time PCR for glyceraldehyde 3-phosphate dehydrogenase (GAPDH) showed a mean (±SD) GAPDH Ct of 26.7 (±2.3), ranging from 23.6 to 34.3, and real-time PCR for ACE-2 showed a mean (±SD) ACE-2 Ct of 32.0 (±2.2), ranging from 28.9 to 37 (Figure 1).

**Figure 2:**
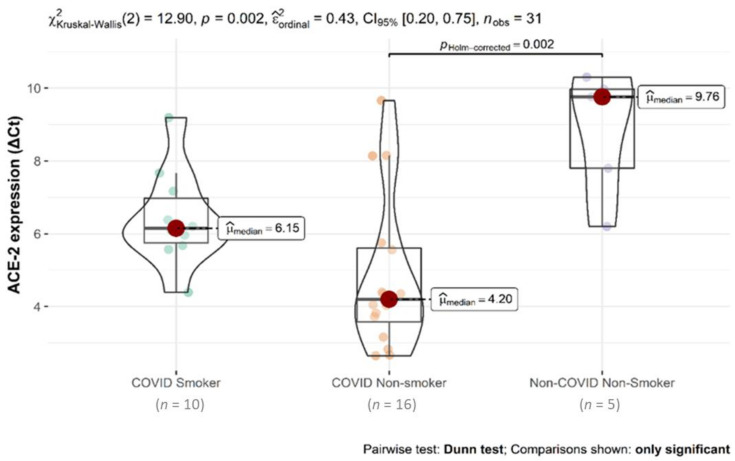
Box-violin plots of ACE-2 expression comparison in the three groups of patients. Overall comparison was implemented with the Kruskal–Wallis test. Pairwise comparisons with the Dunn post-hoc test. Only the COVID non-smokers vs. non-COVID non-smokers comparison was significant (*p* = 0.002). ACE-2 ΔCts (Ct_ACE-2_ − Ct_GAPDH_) are inversely correlated with ACE-2 expression.

Median (Q1–Q3) values of ACE-2 ΔCt were 5.6 (3.8–6.4), ranging from 1.7 to 9.7. Based on median ACE-2 ΔCt value, patients were divided into “high expressors” (ΔCt < 5.6, 16 patients) or “low expressors” (ΔCt ≥ 5.6, 13 patients). Donor lung tissues showed an ACE-2 ΔCt median (Q1–Q3) value of 9.8 (7.8–10), ranging from 6.2 to 10.3; thus, ACE-2 expression was increased 18-fold in cases of SARS-CoV-2 infection (Figure 2).

### 3.3. Correlations between ACE-2 Expression and Clinicopathological Data

**Figure 3:**
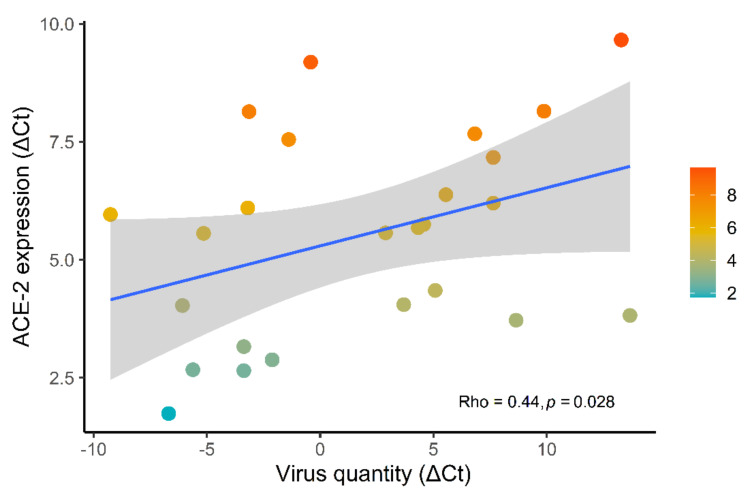
Relation between ACE-2 expression and virus quantity. Direct linear relation between SARS-CoV-2 quantity and ACE-2 receptor expression (*p* = 0.028, rho = 0.44). Shaded areas represent the 95% CI of the linear correlation line.

When comparing N1 and ACE-2 ΔCt, a direct linear relation was found (*p* = 0.028, rho = 0.44): thus, the quantity of SARS-CoV-2 in tissue was directly related to ACE-2 receptor expression (Figure 3).

After dichotomizing ACE-2 expression values, smoking habit was shown to be less frequent in “high expressors” than “low expressors” (14% vs. 67%, *p* = 0.014, Table 2). No other significant differences were found when ACE-2 expression was correlated with the remaining clinical data. The significant effect of ACE-2 expression was also observed when several clinical/pathological data were analyzed in smokers versus non-smokers. Smokers showed a median (IQR) ΔCT of 6.15 (5.75, 6.97) vs. 4.20 (3.58, 5.61) (*p* = 0.012, Table 3). Other clinical parameters did not differ between these two groups of patients.

**Figure 4:**
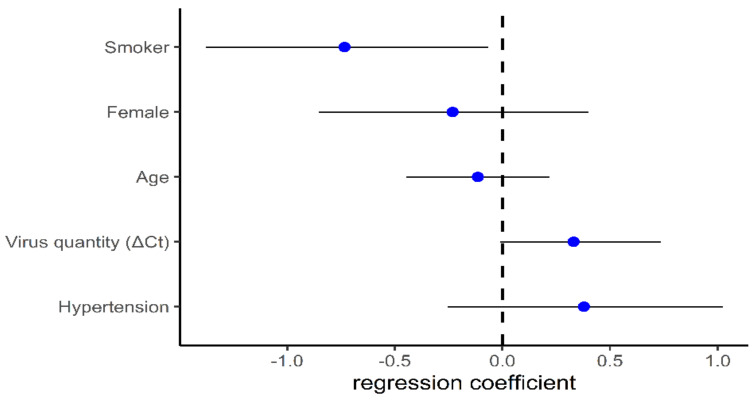
Bayesian linear regression model coefficients. Data are presented as median with 95% highest density interval of the posterior distribution. Coefficients are ranked from the lowest to the highest median.

Bayesian regression model explanatory power is substantial (R^2^ = 0.41, 89% CI [0.24, 0.55], Figure 4). Within this model:

- The effect of smoking (Median = −0.73, 95% CI [−1.41, −0.06]) has a 98.17% probability of being negative (<0), 97.63% of being significant (<−0.05), and 90.42% of being large (<−0.30).
- The effect of age (Median = −0.12, 95% CI [−0.45, 0.22]) has a 76.37% probability of being negative, 65.70% of being significant, and 14.38% of being large.
- The effect of gender (female) (Median = −0.23, 95% CI [−0.90, 0.40]) has a 75.82% probability of being negative, 70.98% of being significant, and 40.55% of being large.
- The effect of hypertension (Median = 0.38, 95% CI [−0.25, 1.03]) has a 88.28% probability of being positive (>0), 84.58% of being significant (>0.05), and 58.97% of being large (>0.30).
- The effect of virus quantity (Median = 0.33, 95% CI [−0.05, 0.72]) has a 95.37% probability of being positive, 92.63% of being significant, and 56.38% of being large.

The estimation successfully converged (R-hat = 1.000) and the indices are reliable (ESS > 7000) for all the variables.

**Figure 5:**
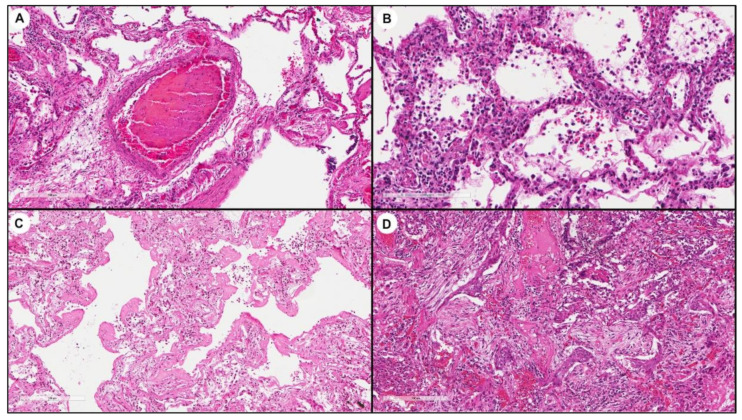
Histological lung sections of an index case with prevalent vascular injury (male, 82 years old, high ACE-2 expressor with ΔCt = 4.4) that shows a thrombus in a centrilobular arteriolar vessel (**A**, hematoxylin and eosin stain, original magnification ×100) and diffuse capillaritis in the alveolar septa (**B**, hematoxylin and eosin stain, original magnification ×200). Histological lung sections of an index case (male, 76 years old, low ACE-2 expressor with ΔCt = 8.2) with diffuse alveolar damage: hyaline membrane deposits along the alveolar walls (**C**, hematoxylin and eosin stain, original magnification ×100), foci of squamous metaplasia associated with endoalveolar exudative material including connective tissue components, fibrin, and fibroblasts (organizing pneumonia) (**D**, hematoxylin and eosin stain, original magnification ×100).

Correlation of real-time PCR data with morphological features showed a significant higher ACE-2 expression in lungs with a prevalent pattern of vascular injury (56% vs. 31%, *p* = 0.049, *q* = 0.3, Table 2, Figure 5).

## 4. Discussion

It is crucial to gain greater understanding of the molecular basis for susceptibility to SARS-CoV-2 infection among different individuals. In particular, it is vital to shed light on the underlying mechanisms of the possible effect of a lifestyle-related factor such as the smoking habit on SARS-CoV-2 infection. 

In the present study, we found low tissue gene expression of the ACE-2 levels in smoker patients with severe COVID-19 pneumonia, independently from other variables such as age, gender, hypertension treatment and virus quantity. The effects of cigarette smoking and SARS-CoV-2 infection-related lung disease are still a matter of debate in the literature.

Epidemiological studies continue to fuel conflicting results. While most of the studies to date have indicated an association between smoking and increased viral infection susceptibility and worsening of COVID-19 symptoms [24,32,33,34,35,36,37,38,39,40,41,42,43,44,45], also highlighting the importance of the cumulative pack-year exposure [41], there are several studies that have observed an inverse relationship between smoking and COVID-19 [44,46,47,48,49,50,51]. In particular, smoking prevalence among patients hospitalized with COVID-19 has been reported to be lower than the smoking prevalence in the general population [46,47,52] and these data were also confirmed in 132 patients who were admitted to our university hospital [53]. 

Several mechanistic studies that focused on more careful investigation of the ACE-2 signaling pathway mainly come from in vitro and/or experimental models reporting contradictory results and ambiguous interpretations as well. Indeed, some authors reported a direct dose-dependent relation between smoking and ACE-2 expression, speculating that this may increase the susceptibility of smokers and/or COPD patients to SARS-CoV-2 infection [25,54,55,56,57,58,59,60]. Indeed, nicotine has been shown to induce airway epithelial expression of the α7 subtype of nicotine acetylcholine receptors (α7-nAChR), which activates MAPK/ERK, resulting in ACE-2 overexpression in the same cells [22,25,61,62,63]. Even if the precise nAChR subunit identification is difficult, others have also been reported associated to smoking (i.e., α5, α10, β2 and β3) [64] and some of them also involved in inflammatory process of lung tissues. Moreover, nicotine increases SARS-CoV-2 replication, transcription of viral proteins, and cytopathic effects [22,62].

However, most of these interesting studies have been performed in cell lines and clinical samples from smokers (with or without COPD) not infected by SARS-CoV-2. Thus, the direct relation between nicotine-induced ACE-2 expression and the susceptibility to the infection was only speculative. 

To the best of our knowledge, the present paper is the first to report a quantification of ACE-2 mRNA expression in the lung tissue from smokers and non-smokers who died due to severe COVID-19 pneumonia.

Several experimental findings are in line with the results of our study. Cigarette smoke (nicotine) may cause structural changes in the ACE-2 genome and this could not only interfere with SARS-CoV-2 spike protein binding [65] but also with regular gene transcription. Nicotine interacts with many components of the RAAS in multiple organs and in multiple organ systems. In the ACE/AT-II (angiotensin II)/AT1R (angiotensin1 receptor) arm, nicotine increases the expression and/or activity of renin, ACE, and AT1R, whereas, in the compensatory ACE-2/angiotensin [6,25,54,55,66,67,68] arm, nicotine downregulates the expression and/or activity of ACE-2 and AT2R [54]. ACE-2 knockout mice exposed to cigarette smoke exhibit increased pulmonary inflammation with activation of metalloproteinases [7] that could, in part, contribute to the inactivation or modification of ACE-2 in the lungs of smokers. A very recent experimental study by Tomchaney et al. showed that mice exposed to cigarette smoke had decreased ACE-2 levels in bronchial and alveolar epithelia. Similarly, cigarette smoke-treatment abrogated SARS-CoV-2 replication in the same cells in vitro [69]. Moreover, although it is possible that cigarette smoke increases ACE-2 expression in the airway, thus facilitating the entry of SARS-CoV-2, this increase does not necessarily translate into higher lung parenchymal expression as was instead found in our study.

In each patient many clinical data, including smoking history, were carefully collected by the physician (at hospital admission) and included in a shared electronic database to avoid overlooking important information. Many of our patients (17 out of 29) with systemic hypertension were treated with anti-hypertensive drugs, which could have had an impact on ACE-2 gene expression. However, multivariable regression analysis showed that hypertension is unlikely to have a large effect on the enzyme expression. 

Also sex differences seem to be implicated in the severity of COVID-19, as men have been shown consistently overrepresented in COVID-19 and associated with severe outcomes, likely due to gender-specific behaviors, genetic and hormonal factors, and sex differences in biological pathways related to SARS-CoV-2 infection [70,71]. However, gender was included as covariate in the regression model but no significant differences were found. 

We found also a direct linear relation between ACE-2 expression and SARS-CoV-2 quantity (*p* = 0.028). In addition, the topic concerning the dynamic interaction between ACE-2 and SARS-CoV-2 is complex and not yet precisely defined. Several previous papers had hypothesized that infection with SARS-CoV results in ACE-2 downregulation through internalization, induced by the binding of SARS-Cov to ACE-2, thus being a process that could contribute to the severity of lung pathology [72,73,74,75]. In contrast, some other studies showed a dramatic increase in ACE-2 after SARS-CoV infection, suggesting a critical role of this receptor in viral susceptibility, in line with our results [76,77]. A few studies have speculated that the high expression of ACE-2 may be related to an immune system dysfunction, particularly influenced by interferon gamma upregulation produced after viral entry [16,78,79,80]. Indeed, it has been shown that ACE-2 is an interferon-induced gene and that ACE-2 levels in airway epithelial cells dramatically increase during SARS-CoV-2 infection [76,77]. However, further studies are urgently needed regarding this complex interplay with focus on both airway and lung parenchyma where the disease is more severe. 

Our finding of high ACE-2 expression in cases with a prevalent morphological pattern of vascular injury is noteworthy. Consistent vascular lesions, mainly with the features of endothelialitis, thrombotic microangiopathy, and pulmonary angiogenesis have been reported as distinctive pathological features of COVID-19 pneumopathy [20,21,22,23,24]. Ackermann et al. showed a great number of ACE-2 positive endothelial cells on lung tissues from COVID-19 autoptic cases, with significant changes in endothelial morphology [81]. Altered endothelial cells and increased vascular bed (neoangiogenesis) in this phenotype could explain its increased level. Moreover, in this phenotype we have consistently demonstrated [8,29] a higher viral quantity than in those with prevalent diffuse alveolar damage. The increased viral quantity could in turn lead to an increase in ACE expression related to the above-mentioned mechanisms.

The present study has some limitations.

In this paper, we have described the expression of ACE-2 transcripts in mammalian lungs, but our study does not guarantee that stimuli affecting the levels of ACE-2 mRNA will have the same effect on the ACE-2 protein. Several papers have documented a strong correlation between ACE-2 mRNA and protein levels [58,82,83], but one cannot exclude that ACE-2 protein is differentially expressed in the lung parenchyma. Unfortunately, the antibodies against ACE-2 do not work in a sensitive and reproducible way.

We did not analyze upper airway tissues, which are also infected with SARS-CoV-2 and represent the first cellular targets. Future studies in airway and lung parenchyma in the same patient are ongoing and could be of extreme value to map the complex interaction ACE-2/SARS-CoV-2 in different anatomic districts.

Even if the deceased patients had a COVID-19 pneumonia of different clinical duration and severity, they can surely represent another clinical phenotype compared to asymptomatic/pauci-symptomatic or mild forms of COVID-19 pneumonia. Although some studies have shown the possibility of carrying out biopsies with minimal risk of viral transmission [84], invasive diagnostic procedures involving whatever type of lung sampling have been discouraged in most centers during the SARSCoV-2 pandemic, including ours.

Another point is that some clinical information was not available, such as cumulative pack-year exposure and other drug abuse; thus, the potential role of these in the ACE-2 expression has not been investigated. However, as recently highlighted in a research letter published in JAMA internal medicine, smoking is sometimes imperfectly classified in medical records and former smokers are potentially classified as never smokers; moreover, pack-years may be under recorded [41]. Moreover, it is unlikely that our patients (with a median age of 82 years old) were drug abusers. 

In addition, most patients were more than 75 years old; thus, the absence of relation between ACE-2 expression and age should be confirmed in larger case series, including younger patients, even if severe forms of COVID-19 rarely occur at this age. Also, the expression of TMPRSS2, a serine protease that allows the fusion of viral and cell membranes, should be quantified, for a better understanding of the SARS-CoV-2 entry process.

ACE-2 is one of the principal receptors, however several lesser known receptors and mediators may be involved in regulating infection risk among individuals, in particular in smoker patients. High throughput approaches such as transcriptomic/proteomic could help to better understand this complex biological interaction.

## 5. Conclusions

A crucial aspect of ongoing efforts to control and mitigate the impact of the COVID-19 pandemic has been to reduce viral transmission among individuals. One of the most important objectives is to gain greater understanding of the modes of transmission, including a more in-depth knowledge of the molecular mechanisms for different susceptibility to infection. 

In summary, we found lower mRNA levels of ACE-2 in lung parenchyma of smoker compared to non-smoker patients with severe COVID-19 pneumonia. We also found a high expression of ACE-2 in patients with high viral quantity and more frequent vascular phenotype. 

Our data could support epidemiological findings reporting a higher prevalence of SARS-CoV-2 infection in non-smokers, but do not give any information about the severity of the disease. Indeed, despite a lower predisposition of smokers to COVID-19, their disease course and prognosis could be worst because of several comorbidities that are commonly associated with smoking habit. This aspect merits further and global studies with a more careful collection of smoking history (such as pack-years and type of smoking).

It is incontrovertible that cigarette smoking is detrimental to the lungs, causing severe diseases (e.g., COPD, lung cancer). We would like to be cautious about the messaging surrounding smoking and COVID-19, especially in these fraught times when messages can be easily misinterpreted by the general public.

These results point out complex biological interactions between SARS-CoV-2 and ACE-2, particularly concerning the aspect of smoking habits, and need larger case series and translation studies.

## Figures and Tables

**Table 1:** Clinical characteristics of the study population.

Characteristic	*N* = 29 ^1^
Age (years)	82 (75–87)
Gender	
Male	17 (59%)
Female	12 (41%)
Smokers	10 (38%)
(Missing)	3
Comorbidities (overall)	28 (97%)
Cardiovascular comorbidities	25 (86%)
Hypertension	17 (59%)
Other infections	11 (38%)
Symptoms (overall)	
Yes	29 (100%)
Cough	17 (59%)
Dyspnea	26 (90%)
Fever	21 (78%)
(Missing)	2
Intensive care unit admission	13 (45%)
Disease duration (days)	9 (7–22)

^1^ Median (IQR); n (%).

**Table 2:** Clinical and morphological characteristics of patients with high vs. low ACE-2 expression.

Characteristic	High ACE-2 Expressors (ΔCt < 5.6), *N* = 16 ^1^	Low ACE-2 Expressors (ΔCt ≥ 5.6), *N* = 13 ^1^	*p*-Value ^2^	*q*-Value ^3^
Age (years)	86 (77–90)	79 (74–83)	0.072	0.3
Age			0.2	0.4
<82years	6 (38%)	8 (62%)		
≥82years	10 (62%)	5 (38%)		
Gender			0.3	0.4
Male	8 (50%)	9 (69%)		
Female	8 (50%)	4 (31%)		
Smokers	2 (14%)	8 (67%)	0.014	0.11
(Missing)	2	1		
ACE-2 expression (ΔCt)	3.92 (2.87–4.37)	7.17 (6.10–8.14)	<0.001	<0.001
Virus quantity (ΔCt)	−2.7 (−5.3–4.0)	4.6 (−1.4–7.6)	0.13	0.4
Disease duration (days)	8 (6–12)	12 (9–24)	0.2	0.4
Comorbidities (overall)	15 (94%)	10 (77%)	0.3	0.4
Hypertension	11 (69%)	6 (46%)	0.2	0.4
Other infections	7 (44%)	4 (31%)	0.7	0.8
Cardiovascular comorbidities	15 (94%)	10 (77%)	0.3	0.4
Morphological diagnosis			0.049	0.3
Vascular	9 (56%)	4 (31%)		
Mixed	7 (44%)	5 (38%)		
ALI	0 (0%)	4 (31%)		
Symptoms (overall)	16 (100%)	13 (100%)		
Cough	9 (56%)	8 (62%)	0.8	0.8
Dyspnea	13 (81%)	13 (100%)	0.2	0.4
Fever	11 (79%)	10 (77%)	>0.9	>0.9
(Missing)	2	0		
Intensive care unit admission	5 (31%)	8 (62%)	0.10	0.3

ACE-2, angiotensin-converting enzyme 2; ALI, acute lung injury; ^1^ Median (IQR); *n* (%); ^2^ Wilcoxon rank sum test; Pearson’s Chi-squared test; Fisher’s exact test; Wilcoxon rank sum exact test; ^3^ False discovery rate correction for multiple testing.

**Table 3:** Clinical and morphological characteristics of smoker vs. non-smoker patients.

Characteristic	Smokers *N* = 10 ^1^	Non-Smokers *N* = 16 ^1^	*p*-Value ^2^	*q*-Value ^3^
Age (years)	80 (77–84)	81 (74–87)	0.9	>0.9
Age			>0.9	>0.9
<82years	5 (50%)	8 (50%)		
≥82years	5 (50%)	8 (50%)		
Gender			0.4	>0.9
Male	8 (80%)	9 (56%)		
Female	2 (20%)	7 (44%)		
ACE-2 expression (ΔCt)	6.15 (5.75–6.97)	4.20 (3.58–5.61)	0.012	0.11
Virus quantity (ΔCt)	4.3 (−0.4–6.8)	3.7 (−3.4–8.7)	>0.9	>0.9
ACE-2 expression			0.014	0.11
High (ΔCt < 5.6)	2 (20%)	12 (75%)		
Low (ΔCt ≥ 5.6)	8 (80%)	4 (25%)		
Disease duration (days)	12 (7–20)	10 (7–24)	>0.9	>0.9
Comorbidities (overall)	9 (90%)	14 (88%)	>0.9	>0.9
Hypertension	5 (50%)	11 (69%)	0.4	>0.9
Other infections	2 (20%)	8 (50%)	0.2	0.9
Cardiovascular comorbidities	9 (90%)	14 (88%)	>0.9	>0.9
Morphological diagnosis			0.4	>0.9
Vascular	4 (40%)	8 (50%)		
Mixed	3 (30%)	7 (44%)		
ALI	3 (30%)	1 (6.2%)		
Symptoms (overall)	10 (100%)	16 (100%)		
Cough	6 (60%)	9 (56%)	>0.9	>0.9
Dyspnea	10 (100%)	14 (88%)	0.5	>0.9
Fever	10 (100%)	11 (73%)	0.12	0.7
(Missing)	0	1		
Intensive care unit admission	5 (50%)	7 (44%)	>0.9	>0.9

^1^ Median (IQR); *n* (%); ^2^ Wilcoxon rank sum test; Pearson’s Chi-squared test; Fisher’s exact test; Wilcoxon rank sum exact test; ^3^ False discovery rate correction for multiple testing; ACE-2, angiotensin converting enzyme 2; ALI, acute lung injury.

## Data Availability

The data and used R code presented in this study are openly available in Zenodo at https://doi.org/10.5281/zenodo.4621047.

## References

[1] Johns Hopkins University Coronavirus Resource Center COVID-19 Dash-Board by the Center for Systems Science and Engineering (CSSE) at Johns Hopkins University. https://coronavirus.jhu.edu/map.html.

[2] Phua J., Weng L., Ling L., Egi M., Lim C.-M., Divatia J.V., Shrestha B.R., Arabi Y.M., Ng J., Gomersall C.D. (2020). Intensive Care Management of Coronavirus Disease 2019 (COVID-19): Challenges and Recommendations. Lancet Respir. Med..

[3] Chakraborty S. (2021). Evolutionary and structural analysis elucidates mutations on SARS-CoV-2 spike protein with altered human ACE-2 binding affinity. Biochem. Biophys. Res. Commun..

[4] Chen J., Wang R., Wang M., Wei G.-W. (2020). Mutations Strengthened SARS-CoV-2 Infectivity. J. Mol. Biol..

[5] Zhou P., Yang X.-L., Wang X.-G., Hu B., Zhang L., Zhang W., Si H.-R., Zhu Y., Li B., Huang C.-L. (2020). A Pneumonia Outbreak Associated with a New Coronavirus of Probable Bat Origin. Nature.

[6] Hoffmann M., Kleine-Weber H., Schroeder S., Krüger N., Herrler T., Erichsen S., Schiergens T.S., Herrler G., Wu N.-H., Nitsche A. (2020). SARS-CoV-2 Cell Entry Depends on ACE-2 and TMPRSS2 and Is Blocked by a Clinically Proven Protease Inhibitor. Cell.

[7] Hamming I., Timens W., Bulthuis M.L.C., Lely A.T., Navis G.J., van Goor H. (2004). Tissue Distribution of ACE-2 Protein, the Functional Receptor for SARS Coronavirus. A First Step in Understanding SARS Pathogenesis. J. Pathol..

[8] Borczuk A.C., Salvatore S.P., Seshan S.V., Patel S.S., Bussel J.B., Mostyka M., Elsoukkary S., He B., Del Vecchio C., Fortarezza F. (2020). COVID-19 Pulmonary Pathology: A Multi-Institutional Autopsy Cohort from Italy and New York City. Mod. Pathol..

[9] Calabrese F., Fortarezza F., Giraudo C., Pezzuto F., Faccioli E., Rea F., Pittarello D., Correale C., Navalesi P. (2020). Two Sorts of Microthrombi in a Patient with Coronavirus Disease 2019 and Lung Cancer. J. Thorac. Oncol..

[10] Gupta A., Madhavan M.V., Sehgal K., Nair N., Mahajan S., Sehrawat T.S., Bikdeli B., Ahluwalia N., Ausiello J.C., Wan E.Y. (2020). Extrapulmonary Manifestations of COVID-19. Nat. Med..

[11] Jia H.P., Look D.C., Shi L., Hickey M., Pewe L., Netland J., Farzan M., Wohlford-Lenane C., Perlman S., McCray P.B. (2005). ACE-2 Receptor Expression and Severe Acute Respiratory Syndrome Coronavirus Infection Depend on Differentiation of Human Airway Epithelia. J. Virol..

[12] Xu H., Zhong L., Deng J., Peng J., Dan H., Zeng X., Li T., Chen Q. (2020). High Expression of ACE-2 Receptor of 2019-NCoV on the Epithelial Cells of Oral Mucosa. Int. J. Oral Sci..

[13] Magrone T., Magrone M., Jirillo E. (2020). Focus on Receptors for Coronaviruses with Special Reference to Angiotensin—Converting Enzyme 2 as a Potential Drug Target—A Perspective. Endocr. Metab. Immune Disord. Drug Targets.

[14] Li J., Gao J., Xu Y.P., Zhou T.L., Jin Y.Y., Lou J.N. (2007). Expression of severe acute respiratory syndrome coronavirus receptors, ACE-2 and CD209L in different organ derived microvascular endothelial cells. Zhonghua Yi Xue Za Zhi.

[15] Hung Y.H., Hsieh W.Y., Hsieh J.S., Liu F.C., Tsai C.H., Lu L.C., Huang C.Y., Wu C.L., Lin C.S. (2016). Alternative Roles of STAT3 and MAPK Signaling Pathways in the MMPs Activation and Progression of Lung Injury Induced by Cigarette Smoke Exposure in ACE-2 Knockout Mice. Int. J. Biol. Sci..

[16] Nawijn M.C., Timens W. (2020). Can ACE 2 expression explain SARS -CoV-2 infection of the respiratory epithelia in COVID -19?. Mol. Syst. Biol..

[17] Ortiz M.E., Thurman A., Pezzulo A.A., Leidinger M.R., Klesney-Tait J.A., Karp P.H., Tan P., Wohlford-Lenane C., McCray P.B., Meyerholz D.K. (2020). Heterogeneous Expression of the SARS-Coronavirus-2 Receptor ACE-2 in the Human Respiratory Tract. EBioMedicine.

[18] Su H., Yang M., Wan C., Yi L.-X., Tang F., Zhu H.-Y., Yi F., Yang H.-C., Fogo A.B., Nie X. (2020). Renal Histopathological Analysis of 26 Postmortem Findings of Patients with COVID-19 in China. Kidney Int..

[19] Bourgonje A.R., Abdulle A.E., Timens W., Hillebrands J.-L., Navis G.J., Gordijn S.J., Bolling M.C., Dijkstra G., Voors A.A., Osterhaus A.D. (2020). Angiotensin-Converting Enzyme 2 (ACE-2), SARS-CoV-2 and the Pathophysiology of Coronavirus Disease 2019 (COVID-19). J. Pathol..

[20] Leung J.M., Sin D.D. (2020). Smoking, ACE-2 and COVID-19: Ongoing Controversies. Eur. Respir. J..

[21] Farsalinos K., Angelopoulou A., Alexandris N., Poulas K. (2020). COVID-19 and the Nicotinic Cholinergic System. Eur. Respir. J..

[22] Russo P., Bonassi S., Giacconi R., Malavolta M., Tomino C., Maggi F. (2020). COVID-19 and Smoking: Is Nicotine the Hidden Link?. Eur. Respir. J..

[23] Mehra M.R., Desai S.S., Kuy S., Henry T.D., Patel A.N. (2020). Cardiovascular Disease, Drug Therapy, and Mortality in Covid-19. N. Engl. J. Med..

[24] Alqahtani J.S., Oyelade T., Aldhahir A.M., Alghamdi S.M., Almehmadi M., Alqahtani A.S., Quaderi S., Mandal S., Hurst J.R. (2020). Prevalence, Severity and Mortality Associated with COPD and Smoking in Patients with COVID-19: A Rapid Systematic Review and Meta-Analysis. PLoS ONE.

[25] Leung J.M., Yang C.X., Tam A., Shaipanich T., Hackett T.-L., Singhera G.K., Dorscheid D.R., Sin D.D. (2020). ACE-2 Expression in the Small Airway Epithelia of Smokers and COPD Patients: Implications for COVID-19. Eur. Respir. J..

[26] Basso C., Calabrese F., Sbaraglia M., Del Vecchio C., Carretta G., Saieva A., Donato D., Flor L., Crisanti A., Dei Tos A.P. (2020). Feasibility of postmortem examination in the era of COVID-19 pandemic: The experience of a Northeast Italy University Hospital. Virchows Arch..

[27] World Health Organization (2020). Clinical Management of Severe Acute Respiratory Infection when Novel Coronavirus (nCoV) Infection is Suspected. Who. https://www.who.int/publications/i/item/10665-332299.

[28] Calabrese F., Pezzuto F., Fortarezza F., Hofman P., Kern I., Panizo A., von der Thüsen J., Timofeev S., Gorkiewicz G., Lunardi F. (2020). Pulmonary pathology and COVID-19: Lessons from autopsy. The experience of European Pulmonary Pathologists. Virchows Arch..

[29] Calabrese F., Pezzuto F., Fortarezza F., Boscolo A., Lunardi F., Giraudo C., Cattelan A., Del Vecchio C., Lorenzoni G., Vedovelli L. (2021). Machine learning-based analysis of alveolar and vascular injury in SARS-CoV-2 acute respiratory failure. J. Pathol..

[30] Lu X., Wang L., Sakthivel S.K., Whitaker B., Murray J., Kamili S., Lynch B., Malapati L., Burke S.A., Harcourt J. (2020). US CDC Real-Time Reverse Transcription PCR Panel for Detection of Severe Acute Respiratory Syndrome Coronavirus 2. Emerg. Infect. Dis..

[31] R Core Team (2021). R: A Language and Environment for Statistical Computing.

[32] Liu W., Tao Z.W., Wang L., Yuan M.L., Liu K., Zhou L., Wei S., Deng Y., Liu J., Liu H.G. (2020). Analysis of factors associated with disease outcomes in hospitalized patients with 2019 novel coronavirus disease. Chin. Med. J..

[33] Guan W.J., Ni Z.Y., Hu Y., Liang W.H., Ou C.Q., He J.X., Liu L., Shan H., Lei C.L., Hui D.S.C. (2020). China Medical Treatment Expert Group for Covid-19. Clinical Characteristics of Coronavirus Disease 2019 in China. N. Engl. J. Med..

[34] Zhang J.J., Dong X., Cao Y.Y., Yuan Y.D., Yang Y.B., Yan Y.Q., Akdis C.A., Gao Y.D. (2020). Clinical characteristics of 140 patients infected with SARS-CoV-2 in Wuhan, China. Allergy.

[35] Zhou F., Yu T., Du R., Fan G., Liu Y., Liu Z., Xiang J., Wang Y., Song B., Gu X. (2020). Clinical course and risk factors for mortality of adult inpatients with COVID-19 in Wuhan, China: A retrospective cohort study. Lancet.

[36] Yu T., Cai S., Zheng Z., Cai X., Liu Y., Yin S., Peng J., Xu X. (2020). Association Between Clinical Manifestations and Prognosis in Patients with COVID-19. Clin. Ther..

[37] Patanavanich R., Glantz S.A. (2020). Smoking Is Associated With COVID-19 Progression: A Meta-analysis. Nicotine Tob. Res..

[38] Zhao Q., Meng M., Kumar R., Wu Y., Huang J., Lian N., Deng Y., Lin S. (2020). The impact of COPD and smoking history on the severity of COVID-19: A systemic review and meta-analysis. J. Med. Virol..

[39] Zheng Z., Peng F., Xu B., Zhao J., Liu H., Peng J., Li Q., Jiang C., Zhou Y., Liu S. (2020). Risk factors of critical & mortal COVID-19 cases: A systematic literature review and meta-analysis. J. Infect..

[40] Guo F.R. (2020). Active smoking is associated with severity of coronavirus disease 2019 (COVID-19): An update of a meta-analysis. Tob. Induc. Dis..

[41] Lowe K.E., Zein J., Hatipoglu U., Attaway A. (2021). Association of Smoking and Cumulative Pack-Year Exposure With COVID-19 Outcomes in the Cleveland Clinic COVID-19 Registry. JAMA Intern. Med..

[42] Zhang H., Ma S., Han T., Qu G., Cheng C., Uy J.P., Shaikh M.B., Zhou Q., Song E.J., Sun C. (2021). Association of smoking history with severe and critical outcome in COVID-19 patients: A systemic review and meta-analysis. Eur. J. Integr. Med..

[43] Umnuaypornlert A., Kanchanasurakit S., Lucero-Prisno D.E.I., Saokaew S. (2021). Smoking and risk of negative outcomes among COVID-19 patients: A systematic review and meta-analysis. Tob. Induc. Dis..

[44] Hopkinson N.S., Rossi N., El-Sayed M.J., Laverty A.A., Quint J.K., Freidin M., Visconti A., Murray B., Modat M., Ourselin S. (2021). Current smoking and COVID-19 risk: Results from a population symptom app in over 2.4 million people. Thorax.

[45] Monteiro A.C., Suri R., Emeruwa I.O., Stretch R.J., Cortes-Lopez R.Y., Sherman A., Lindsay C.C., Fulcher J.A., Goodman-Meza D., Sapru A. (2020). Obesity and smoking as risk factors for invasive mechanical ventilation in COVID-19: A retrospective, observational cohort study. PLoS ONE.

[46] Farsalinos K., Barbouni A., Niaura R. (2021). Systematic review of the prevalence of current smoking among hospitalized COVID-19 patients in China: Could nicotine be a therapeutic option? Reply. Intern. Emerg. Med..

[47] Emami A., Javanmardi F., Pirbonyeh N., Akbari A. (2020). Prevalence of Underlying Diseases in Hospitalized Patients with COVID-19: A Systematic Review and Meta-Analysis. Arch. Acad. Emerg. Med..

[48] Petrilli C.M., Jones S.A., Yang J., Rajagopalan H., O’Donnell L., Chernyak Y., Tobin K.A., Cerfolio R.J., Francois F., Horwitz L.I. (2020). Factors associated with hospital admission and critical illness among 5279 people with coronavirus disease 2019 in New York City: Prospective cohort study. BMJ.

[49] Lippi G., Henry B.M. (2020). Active smoking is not associated with severity of coronavirus disease 2019 (COVID-19). Eur. J. Intern. Med..

[50] Farsalinos K., Bagos P.G., Giannouchos T., Niaura R., Barbouni A., Poulas K. (2021). Smoking prevalence among hospitalized COVID-19 patients and its association with disease severity and mortality: An expanded re-analysis of a recent publication. Harm Reduct. J..

[51] Paleiron N., Mayet A., Marbac V., Perisse A., Barazzutti H., Brocq F.X., Janvier F., Bertrand D., Bylicki O. (2021). Impact of Tobacco Smoking on the risk of COVID-19. A large scale retrospective cohort study. Nicotine Tob. Res..

[52] Shastri M.D., Shukla S.D., Chong W.C., Kc R., Dua K., Patel R.P., Peterson G.M., O’Toole R.F. (2020). Smoking and COVID-19: What we know so far. Respir. Med..

[53] Rossato M., Russo L., Mazzocut S., Di Vincenzo A., Fioretto P., Vettor R. (2020). Current smoking is not associated with COVID-19. Eur. Respir. J..

[54] Cai G., Bossé Y., Xiao F., Kheradmand F., Amos C.I. (2020). Tobacco smoking increases the lung gene expression of ACE-2, the receptor of SARS-CoV-2. Am. J. Respir. Crit. Care Med..

[55] Zhang H., Rostami M.R., Leopold P.L., Mezey J.G., O’Beirne S.L., Strulovici-Barel Y., Crystal R.G. (2020). Expression of the SARS-CoV-2 ACE-2 Receptor in the Human Airway Epithelium. Am. J. Respir. Crit. Care Med..

[56] Jacobs M., Van Eeckhoutte H.P., Wijnant S.R.A., Janssens W., Joos G.F., Brusselle G.G., Bracke K.R. (2020). Increased expression of ACE-2, the SARS-CoV-2 entry receptor, in alveolar and bronchial epithelium of smokers and COPD subjects. Eur. Respir. J..

[57] Smith J.C., Sausville E.L., Girish V., Yuan M.L., Vasudevan A., John K.M., Sheltzer J.M. (2020). Cigarette Smoke Exposure and Inflammatory Signaling Increase the Expression of the SARS-CoV-2 Receptor ACE-2 in the Respiratory Tract. Dev. Cell.

[58] Aguiar J.A., Tremblay B.J., Mansfield M.J., Woody O., Lobb B., Banerjee A., Chandiramohan A., Tiessen N., Cao Q., Dvorkin-Gheva A. (2020). Gene expression and in situ protein profiling of candidate SARS-CoV-2 receptors in human airway epithelial cells and lung tissue. Eur. Respir. J..

[59] Sifat A.E., Nozohouri S., Villalba H., Vaidya B., Abbruscato T.J. (2020). The Role of Smoking and Nicotine in the Transmission and Pathogenesis of COVID-19. J. Pharmacol. Exp. Ther..

[60] Liu A., Zhang X., Li R., Zheng M., Yang S., Dai L., Wu A., Hu C., Huang Y., Xie M. (2021). Overexpression of the SARS-CoV-2 receptor ACE2 is induced by cigarette smoke in bronchial and alveolar epithelia. J. Pathol..

[61] Tizabi Y., Getachew B., Copeland R.L., Aschner M. (2020). Nicotine and the nicotinic cholinergic system in COVID-19. FEBS J..

[62] Lupacchini L., Maggi F., Tomino C., De Dominicis C., Mollinari C., Fini M., Bonassi S., Merlo D., Russo P. (2020). Nicotine Changes Airway Epithelial Phenotype and May Increase the SARS-COV-2 Infection Severity. Molecules.

[63] Olds J.L., Kabbani N. (2020). Is nicotine exposure linked to cardiopulmonary vulnerability to COVID-19 in the general population?. FEBS J..

[64] Diabasana Z., Perotin J.M., Belgacemi R., Ancel J., Mulette P., Delepine G., Gosset P., Maskos U., Polette M., Deslée G. (2020). Nicotinic Receptor Subunits Atlas in the Adult Human Lung. Int. J. Mol. Sci..

[65] Hussain M., Jabeen N., Raza F., Shabbir S., Baig A.A., Amanullah A., Aziz B. (2020). Structural variations in human ACE-2 may influence its binding with SARS-CoV-2 spike protein. J. Med. Virol..

[66] Sungnak W., Huang N., Bécavin C., Berg M., Queen R., Litvinukova M., Talavera-López C., Maatz H., Reichart D., Sampaziotis F. (2020). SARS-CoV-2 entry factors are highly expressed in nasal epithelial cells together with innate immune genes. Nat. Med..

[67] Hou Y.J., Okuda K., Edwards C.E., Martinez D.R., Asakura T., Dinnon K.H., Kato T., Lee R.E., Yount B.L., Mascenik T.M. (2020). SARS-CoV-2 Reverse Genetics Reveals a Variable Infection Gradient in the Respiratory Tract. Cell.

[68] Polverino F. (2020). Cigarette Smoking and COVID-19: A Complex Interaction. Am. J. Respir. Crit. Care Med..

[69] Tomchaney M., Contoli M., Mayo J., Baraldo S., Shuaizhi L., Cabel C.R., Bull D.A., Lick S., Malo J., Knoper S. (2020). Paradoxical effects of cigarette smoke and COPD on SARS-CoV2 infection and disease. bioRxiv.

[70] Haitao T., Vermunt J.V., Abeykoon J., Ghamrawi R., Gunaratne M., Jayachandran M., Narang K., Parashuram S., Suvakov S., Garovic V.D. (2020). COVID-19 and Sex Differences: Mechanisms and Biomarkers. Mayo Clin. Proc..

[71] Viveiros A., Rasmuson J., Vu J., Mulvagh S.L., Yip C.Y.Y., Norris C.M., Oudit G.Y. (2021). Sex differences in COVID-19: Candidate pathways, genetics of ACE2, and sex hormones. Am. J. Physiol. Heart Circ. Physiol..

[72] Wang S., Guo F., Liu K., Wang H., Rao S., Yang P., Jiang C. (2008). Endocytosis of the receptor-binding domain of SARS-CoV spike protein together with virus receptor ACE-2. Virus Res..

[73] Kuba K., Imai Y., Rao S., Gao H., Guo F., Guan B., Huan Y., Yang P., Zhang Y., Deng W. (2005). A crucial role of angiotensin converting enzyme 2 (ACE2) in SARS coronavirus-induced lung injury. Nat. Med..

[74] Oudit G.Y., Kassiri Z., Jiang C., Liu P.P., Poutanen S.M., Penninger J.M., Butany J. (2009). SARS-coronavirus modulation of myocardial ACE2 expression and inflammation in patients with SARS. Eur. J. Clin. Investig..

[75] Glowacka I., Bertram S., Herzog P., Pfefferle S., Steffen I., Muench M.O., Simmons G., Hofmann H., Kuri T., Weber F. (2010). Differential downregulation of ACE2 by the spike proteins of severe acute respiratory syndrome coronavirus and human coronavirus NL63. J. Virol..

[76] Li G., He X., Zhang L., Ran Q., Wang J., Xiong A., Wu D., Chen F., Sun J., Chang C. (2020). Assessing ACE-2 expression patterns in lung tissues in the pathogenesis of COVID-19. J. Autoimmun..

[77] Chua R.L., Lukassen S., Trump S., Hennig B.P., Wendisch D., Pott F., Debnath O., Thürmann L., Kurth F., Völker M.T. (2020). COVID-19 severity correlates with airway epithelium-immune cell interactio.s identified by single-cell analysis. Nat. Biotechnol..

[78] Rockx B., Baas T., Zornetzer G.A., Haagmans B., Sheahan T., Frieman M., Dyer M.D., Teal T.H., Proll S., van den Brand J. (2009). Early upregulation of acute respiratory distress syndrome-associated cytokines promotes lethal disease in an aged-mouse model of severe acute respiratory syndrome coronavirus infection. J. Virol..

[79] He L., Ding Y., Zhang Q., Che X., He Y., Shen H., Wang H., Li Z., Zhao L., Geng J. (2006). Expression of elevated levels of pro-inflammatory cytokines in SARS-CoV-infected ACE-2+ cells in SARS patients: Relation to the acute lung injury and pathogenesis of SARS. J. Pathol..

[80] Ziegler C.G.K., Allon S.J., Nyquist S.K., Mbano I.M., Miao V.N., Tzouanas C.N., Cao Y., Yousif A.S., Bals J., Hauser B.M. (2020). SARS-CoV-2 Receptor ACE-2 Is an Interferon-Stimulated Gene in Human Airway Epithelial Cells and Is Detected in Specific Cell Subsets across Tissues. Cell.

[81] Ackermann M., Verleden S.E., Kuehnel M., Haverich A., Welte T., Laenger F., Vanstapel A., Werlein C., Stark H., Tzankov A. (2020). Pulmonary Vascular Endothelialitis, Thrombosis, and Angiogenesis in Covid-19. N. Engl. J. Med..

[82] Wang Y., Wang Y., Luo W., Huang L., Xiao J., Li F., Qin S., Song X., Wu Y., Zeng Q. (2020). A comprehensive investigation of the mRNA and protein level of ACE-2, the putative receptor of SARS-CoV-2, in human tissues and blood cells. Int. J. Med. Sci..

[83] Hikmet F., Méar L., Edvinsson Å., Micke P., Uhlén M., Lindskog C. (2020). The protein expression profile of ACE-2 in human tissues. Mol. Syst. Biol..

[84] Luo F., Darwiche K., Singh S., Torrego A., Steinfort D.P., Gasparini S., Liu D., Zhang W., Fernandez-Bussy S., Herth F.J.F. (2020). Performing Bronchoscopy in Times of the COVID-19 Pandemic: Practice Statement from an International Expert Panel. Respiration.

